# High loss to follow-up on a bedaquiline-based regimen for rifampicin-resistant TB

**DOI:** 10.5588/ijtldopen.25.0261

**Published:** 2025-11-12

**Authors:** F. Idowu, T. Decroo, A. Mesic

**Affiliations:** 1 Department of Public Health, Federal Ministry of Health, Abuja, FCT, Nigeria;; 2 Department of Clinical Sciences, Institute of Tropical Medicine, Antwerp, Belgium.

**Keywords:** tuberculosis, Southwest Zone, Nigeria, LTFU, acquired bedaquiline resistance, treatment outcomes, unfavourable outcomes

Dear Editor,

TB is a public health challenge in Nigeria, one of the top 30 high-burden countries.^1^ An estimated 499,000 new TB cases and 9,400 rifampicin-resistant TB (RR-TB) cases occur in Nigeria each year.^1^ Bedaquiline (BDQ) has been used for RR-TB treatment since 2019.^2^ Despite this short period, BDQ resistance is on the rise and baseline BDQ resistance is observed among 2.4% (95% CI, 1.7–3.5) of BDQ-naïve people with RR-TB. Acquired BDQ resistance is reported in 2.1% (95% CI, 1.4–3.0) of persons receiving BDQ-containing treatment.^3^ BDQ has a long half-life (5.5 months),^2^ therefore, loss to follow-up (LTFU) before achieving culture conversion poses a risk of acquired BDQ resistance. Nigeria commenced the use of the 9-month all-oral BDQ-based regimen (bedaquiline, moxifloxacin, clofazimine, high-dose isoniazid, prothionamide, ethambutol and pyrazinamide) in 2020.^2^ Since November 2023, the six-month BPaLM regimen (bedaquiline, pretomanid, linezolid, and moxifloxacin) has become the preferred option for eligible RR-TB cases.^2^ A previous study from Nigeria among persons on RR-TB treatment, with the majority on injectables, revealed a high proportion of deaths (22/141, 15.6%) and LTFU (15/141, 10.6%).^4^ No previous study evaluated the treatment outcomes of the 9-month all-oral BDQ-based regimen in Nigeria. Considering the high LTFU with previous treatment regimens and the potentially detrimental effect of LTFU on BDQ’s longevity, our study assessed the treatment outcomes and factors associated with unfavourable outcomes among RR-TB patients treated with the 9-month all-oral BDQ-based regimen in Nigeria.

We conducted a retrospective cohort study utilising routinely collected aggregate data of 650 persons with RR-TB enrolled in care in 2022 in the Southwest Zone, Nigeria. 19 out of a cohort of 650 (3%) that commenced RR-TB treatments were excluded from our study. 595 (92%) participants were treated with the 9-month all-oral BDQ-based regimen. 55 (8%) participants were treated with a 9-month operational research regimen (bedaquiline, levofloxacin, linezolid, and clofazimine) and could not be analysed separately, given the use of aggregate data. Treatment outcomes were defined as in the national and WHO guidelines.^5^ Crude odds and adjusted odds ratios were estimated for unfavourable treatment outcomes (death, LTFU, or treatment failure) and LTFU using logistic regression for aggregate data (blogit; Stata: Release 18. College Station, TX: StataCorp LLC). Ethical approval was obtained from the National Health Research Ethics Committee of Nigeria (NHREC).

A total of 631 participants were enrolled in our study. Treatment success was achieved among 454 (71.9%) participants; 295 (46.8%) were cured, and 159 (25.2%) completed treatment. Death was observed among 46 (7.3%), LTFU among 126 (20.0%), and treatment failure among 5 (0.8%) participants. Treatment success was similar to the global average of 68% in 2021,^1^ but lower than the 84% national average reported in 2021.^1^ The odds of having an unfavourable treatment outcome were significantly higher in males compared to females (aOR 1.74, 95% CI: 1.19–2.55, p=0.004; Table 1), which is consistent with previous research.^6^ Delayed diagnosis/treatment, work obligations and challenges in adhering to clinic visits could play a role.^7^ Among the 631 participants enrolled, 28 (4.4%) were HIV-positive. HIV status was strongly associated with unfavourable treatment outcomes (aOR 3.02, 95% CI: 1.44–6.30, p=0.003; Table 1), aligning with previous research.^6^ Opportunistic infections, pill burden, and increased mortality risk are potential explanations.

**Table.** Factors associated with unfavourable treatment outcomes and loss to follow-up in persons with rifampicin-resistant TB in Southwest Nigeria.

	Favorable**^A^** N (%)	Unfavorable**^B^** N (%)	Adjusted OR (95% CI)	Retained**^C^** N (%)	LTFU**^D^** N (%)	Adjusted OR (95% CI)
Total	454 (71.9)	177 (28.1)	NA	505 (80.0)	126 (20.0)	NA
Age						
0–14 yrs	25 (80.6)	6 (19.4)	0.56 (0.22–1.44)	30 (96.8)	1 (3.2)	0.12 (0.02–0.89)**^E^**
≥15 yrs	429 (71.5)	171 (28.5)	1	475 (79.2)	125 (20.8)	1
Gender						
Male	263 (68.1)	123 (31.9)	1.74 (1.19–2.55)*	302 (78.2)	84 (21.8)	1.36 (0.90–2.06)
Female	191 (78.0)	54 (22.0)	1	203 (82.9)	42 (17.1)	1
HIV status						
HIV Positive	15 (53.6)	13 (46.4)	3.02 (1.44–6.30)*	20 (71.4)	8 (28.6)	1.84 (0.78–4.34)
HIV Negative	439 (72.8)	164 (27.2)	1	485 (80.4)	118 (19.6)	1

A Favourable treatment outcomes include cured (N=295) and treatment completed (N=159).

B Unfavourable treatment outcomes include death (N=46), lost to follow-up (N=126) and treatment failure (N=5).

C Retained: retained in care, includes treatment success (cured and treatment completed), death and treatment failure.

D LTFU = loss to follow-up.

E significant

NA = not applicable; OR = odds ratio; CI = confidence interval.

The concerningly high proportion of LTFU (20.0%) exceeds the global average of 14.3% in 2021, the national average of 5.7% in the same year,^1,8^ and the range of LTFU (4–12.7%) reported in previous studies from Nigeria. ^4,9,10^ Nwonsu et al. reported that RR-TB treatment outcomes in Nigeria vary from one geographical zone to another.^11^ Previous studies used decentralised community-based care models, including patients’ support groups organised by the community-based organisations and regular meetings at the state level between community-based health care staff and the state TB team on patient management. Although decentralised care and community structures persist, support for the patients’ support groups and shared patient management ceased pre-study and remains inactive in our study region. Financial support to patients was also significantly reduced during the study due to funding constraints; however, it has improved now. Future research should explore whether decentralisation and stronger community support can reduce LTFU in the study setting. Age was significantly associated with LTFU (aOR 0.12, 95% CI: 0.02–0.89, p=0.04), while gender and HIV status were not. Persons with RR-TB who are 0-14 years were 88% less likely to be LTFU compared to those 15 years and above. This should be interpreted with caution due to sparse data in the 0–14 age group that were LTFU, which led to unstable estimates and wide uncertainty despite the large sample size. (Table 1) Other contributing factors to LTFU may include adverse events, poverty, and lack of food.^12^

High LTFU has major implications for patients and communities. Patients who interrupt treatment are at risk of severe disease and increased mortality, and TB may continue to be transmitted.^13^ Moreover, when the BDQ-based regimen is stopped, “BDQ monotherapy” will continue, as BDQ has a much longer half-life than other TB drugs. “BDQ monotherapy” combined with multiplying bacilli in people who interrupted treatment before being cured enhances the risk of acquired BDQ resistance. Indeed, R. Liang et al. showed that 33% of participants who interrupted RR-TB treatment with BDQ-based regimens were culture-positive upon return to care, and 53% of post-interruption culture isolates were resistant to bedaquiline.^14^ Patients with RR-TB, who are also resistant to BDQ, have few therapeutic options.^15^ Transmission of such highly resistant TB is a major public health concern.

This is the first study to describe the outcomes of the 9-month all-oral BDQ-based regimen in Nigeria. The use of aggregate data limited our ability to explore other relevant factors or to make further stratified analyses. The evaluation of the two regimens used in this study could also not be stratified on this account. The small proportion of participants on the operational research regimen probably did not affect outcome estimates shown in our large cohort. A more in-depth assessment should use individual patient data.

In conclusion, our study demonstrated a worryingly high loss to follow-up rate in persons on a bedaquiline-based treatment regimen for RR-TB. This raises serious concerns for acquired bedaquiline resistance, threatening the long-term impact of bedaquiline-based regimens. Urgent actions are needed to address the loss to follow-up. Decentralisation of RR-TB management, strengthened community support,^15^ and adherence support may help improve patient retention in care.
